# Pressure-induced order–disorder transition in Gd_1.5_Ce_0.5_Ti_2_O_7_ pyrochlore

**DOI:** 10.1098/rsos.190842

**Published:** 2019-09-04

**Authors:** Jingjing Niu, Xiang Wu, Haibin Zhang, Shan Qin

**Affiliations:** 1 Key Laboratory of Continental Collision and Plateau Uplift, Institute of Tibetan Plateau Research, Chinese Academy of Sciences, Beijing 100101, People's Republic of China; 2 Key Laboratory of Orogenic Belts and Crustal Evolution, MOE, Peking University and School of Earth and Space Sciences, Peking University, Beijing 100871, People's Republic of China; 3 Innovation Research Team for Advanced Ceramics, Institute of Nuclear Physics and Chemistry, China Academy of Engineering Physics, Mianyang 621900, People's Republic of China; 4 State key laboratory of geological processes and mineral resources, China University of Geosciences (Wuhan), Wuhan 430074, People's Republic of China

**Keywords:** pyrochlore, order–disorder phase transition, high pressure

## Abstract

An experimental study on ordered pyrochlore structured Gd_1.5_Ce_0.5_Ti_2_O_7_ $\left(Fd\bar{3}m\right)$ was carried out up to 45 GPa by synchrotron radiation X-ray diffraction and Raman spectroscopy. Experimental results show that Gd_1.5_Ce_0.5_Ti_2_O_7_ transfers to a disordered cotunnite-like phase (*Pnma* Z = 4) at approximately 42 GPa. Compared with the end member Gd_2_Ti_2_O_7_, the substitution of Ce^3+^ for Gd^3+^ increases the transition pressure and the high-pressure stability of the pyrochlore phase. This pressure-induced structure transition is mainly controlled by cationic order–disorder modification, and the cationic radius ratio *r*_A_/*r*_B_ may also be effective for predicting the pyrochlore oxides' high-pressure stability. Two isostructural transitions are observed at 6.5 GPa and 13 GPa, and the unit-cell volume of Gd_1.5_Ce_0.5_Ti_2_O_7_ as a function of pressure demonstrates its compression behaviour is rather complex.

## 1. Introduction

**Figure 1.**
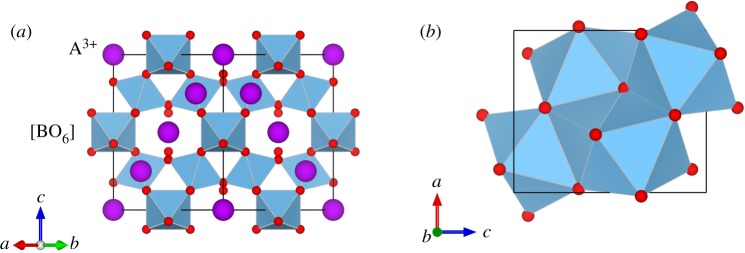
The crystal structures of A_2_B_2_O_7_ oxide: (*a*) the pyrochlore structure and (*b*) the cotunnite-like structure. The purple balls are A^3+^ cations and the blue octahedrons are [BO_6_]. Small red balls are oxygen ions. In the cotunnite-like A_2_B_2_O_7_ structure, the A and B cations are disordered and 1/8 of the O^2−^ vacancies are randomly distributed.

Pyrochlore oxide, with an ideal chemical formula of A_2_B_2_O_6_O'(or A_2_B_2_O_7_), have attracted substantial attention due to its unique structural properties and its applications in fuel cells [1,2], spin liquid materials [3] and high-level waste disposal materials [4]. The pyrochlore structure (figure 1*a*) belongs to the $Fd\bar{3}m$ space group (Z = 8). It can be viewed as A and B cation ordered 2 × 2 × 2 superlattices of ideal fluorite structures (*Fm*3*m*) with 1/8 anion deficiency. The larger A cations occupied 16*c* (1/2 1/2 1/2) with eight coordinates located in a distorted cubic polyhedron. The six-coordinate B site located in 16*d* (0 0 0) is usually occupied by smaller cations centred in an oxygen octahedron. The oxygen O^2−^ anions occupy the 48*f* (*x* 3/8 3/8) site, the O′^2−^ anions occupy the 8*a* (1/8 1/8 1/8) site, and the 8*b* site is systematically vacant. Empirically, the structural stability of A_2_B_2_O_7_ pyrochlores at ambient conditions depends on the ratio of the cation radii, *r*_A_/*r*_B_ [5], only when 1.46 < *r*_A_/*r*_B_ < 1.78, A_2_B_2_O_7_ oxides crystallize in the pyrochlore structure.

As an important thermodynamic parameter, pressure can strongly affect the structures and properties of materials. The safe immobilization of toxic high-level nuclear waste (HLW) requires the waste forms are isolated from biosphere over time scales much longer than the span of recorded human history, because of the radiotoxicity of long half-life isotopes (^239^Pu, half-life 24 000a). Collapse, explosion and geological changes may exert high pressure on the forms of HLW which are enclosed in the disposal repository at a depth of approximately 1000 m underground. Besides, the large radius actinides substituted into the pyrochlore lattice will decrease the phase stability of the lattice under pressure (e.g. pv-ppv transition pressure of NaMgF_3_ and MgSiO_3_ [6]). Above all, the phase stability of the substituted pyrochlore under high pressure needs to be considered.

The high-pressure behaviour of pure member pyrochlore oxides has been extensively investigated. Theoretical simulations reveal that titanate pyrochlores (B = Ti^4+^) and zirconated pyrochlore (B = Zr^4+^) can transfer to an orthorhombic cotunnite-like structure (*Pnma* and Z = 4) (figure 1*b*) at high pressure [7–9]. At 11 GPa and 1300°C, pyrochlore Eu_2_Ti_2_O_7_ transfers to a perovskite-like structure (*P*2_1_, denoted PL-Eu_2_Ti_2_O_7_), which has been confirmed as a high-temperature ferroelectric material [10]. In addition, the substitution of pyrochlore-type oxides can form complex composition pyrochlore oxides, which helps control their structure and physical properties. In the Gd_2_Ti_2−x_Zr_x_O_7_ binary system, the transition pressure increases along with the decrease of the B site substituting Zr^4+^ [11], while the transition pressure slightly changes in zirconated pyrochlore (Gd_0.9_U_0.1_)(Zr_0.9_U_0.1_)O_7+*δ*_ with U-doped in both the A site and B site [12]. Ce^3+^ is often used in research as a nonradioactive surrogate for Pu because they share common chemical and crystal-chemical properties. Zhang *et al*. revealed that the Gd_2−x_Ce_x_Ti_2_O_7_ system maintains pyrochlore structures when *x* < 0.8 [13]. Here, we chose Ce^3+^ substitution for Gd^3+^ to increase the A site average cationic radius, and carried out the experimental study on Gd_1.5_Ce_0.5_Ti_2_O_7_ up to approximately 40 GPa, in order to explore the Ce^3+^-doping influence on its phase stability and high-pressure behaviour.

## 2. Experimental details

The sample in the present study was synthesized using a combustion method. The starting materials tetrabutyl titanate [Ti(OBu)_4_] (Aladdin, greater than 99.0%), Gd(NO_3_)_3_·6H_2_O (Aladdin, 99.9%) and Ce(NO_3_)_3_·6H_2_O (Aladdin, 99.9%), were dissolved stoichiometrically in nitric acid and deionized water, respectively, with magnetic stirring. Glycine (Aladdin, 99%) as a fuel with a mole ratio *n*(Gly)/*n*(Ti) = 2.8 was added to the mixed solution. This mixture was heated on a hot plate until an auto-ignition process in a corundum crucible. The obtained solid was sintered at 1473 K for 2 h under Argon atmosphere in order to avoid the Ce^3+^ being oxidized.

Symmetry-type diamond anvil cells were employed as a high-pressure apparatus. Two runs of *in situ* synchrotron X-ray diffraction experiments were carried out under *λ* = 0.6199 Å. Rhenium gaskets were pre-indented to approximately 40 µm in thickness with a hole approximately 150 µm in diameter in the centre of the indentation as sample chambers. Run 1 was performed at the Shanghai Synchrotron Radiation Facility (SSRF) BL15U1 beamline at a pressure up to approximately 47 GPa. The pressure transmitting media was silicone oil. A slice of Au (99.9%, Alfa Aesar, Haverhill, MA, USA) was loaded into the sample chamber, and its equation of state (EoS) was used to determine the pressure [14]. In run 2, an experiment up to approximately 20 GPa was carried out at the Beijing Synchrotron Radiation Facility (BSRF) 4W2 beamline. Noble gas argon was loaded into the sample chamber as PTM and the pressure was monitored using the ruby fluorescence method [15]. All of the XRD patterns were converted from Debye rings to one-dimensional X-ray profiles versus 2*θ* via FIT2D code [16]. The high-pressure XRD patterns were fitted using the Le Bail method implemented using GSAS + EXPGUI software [17].

High-pressure Raman experiments were carried out up to approximately 40 GPa at room temperature on a Renishaw inVia reflex laser Raman spectrometer. A 532 nm diode-pumped solid-state laser was employed as the excitation light source. The polycrystalline sample was compressed into slices and placed in a 150 µm diameter hole drilled in pre-indented Rhenium gaskets. The ruby fluorescence technique was employed to calibrate the pressure [15], and silicone oil was used as the pressure medium.

## 3. Results

**Figure 2.**
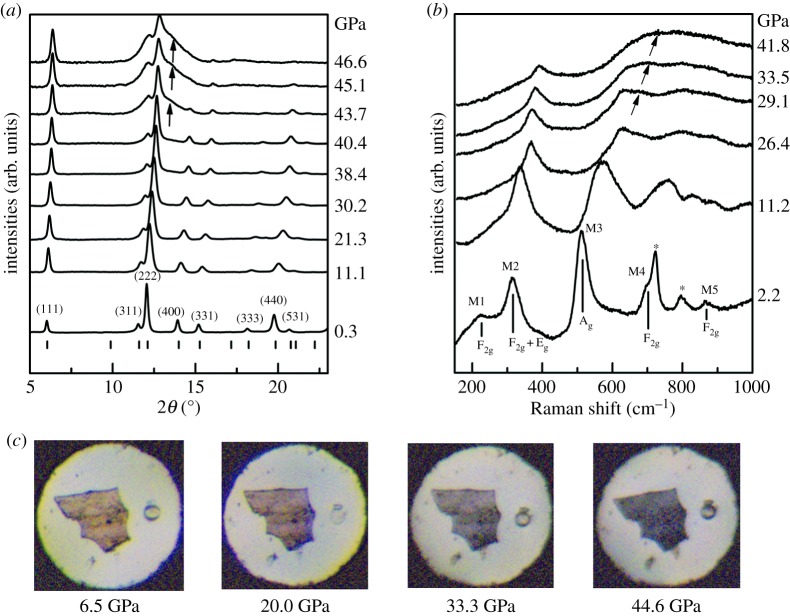
(*a*) The *in situ* synchrotron high-pressure XRD patterns of run 1. The arrow indicates that the new reflections belong to the *Pnma* cotunnite-like phase. (*b*) The *in situ* high-pressure Raman spectra of the Gd_1.5_Ce_0.5_Ti_2_O_7_. The star (*) mode belongs to the silicone oil, and the arrows indicate the new band observed at approximately 700 cm^−1^ when the pressure is higher than 29.1 GPa. (*c*) The sample's colour change under different pressures.

**Figure 3.**
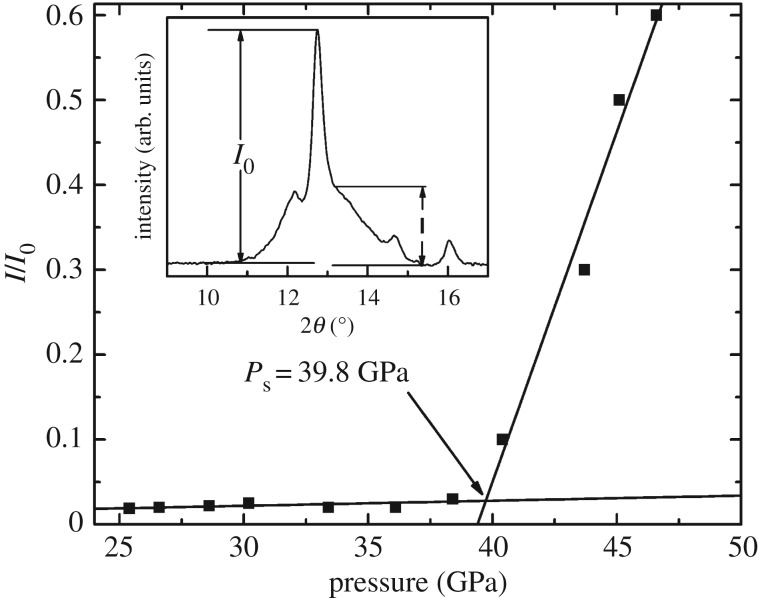
The intensity ratio between (222) of the pyrochlore and cotunnite phase as a function of pressure. Cotunnite intensity is defined as the intensity between the (222) and (004) pyrochlore reflections.

Figure 2*a* shows the selected XRD patterns from run 1 of the Gd_1.5_Ce_0.5_Ti_2_O_7_ under different pressure. The patterns of run 2 are available in the supplementary materials (electronic supplementary material, figure S1). At the beginning of the experiments, all of the reflections can be indexed as a pyrochlore structure, indicating that the Gd_1.5_Ce_0.5_Ti_2_O_7_ crystallized in the pyrochlore structure. At 43.7 GPa, a new reflection arises at approximately 13° between the (222) and (400) reflections of the pyrochlore structure. The intensities of (400), (331), (333), (440) and (531) reflections decrease while the intensity of (111) increases. The sample undergoes a pressure-induced phase transformation to an orthorhombic cotunnite-like phase (*Pnma*). Due to the high degree of disorder and large strain inherent in the high-pressure cotunnite-like phase, it is difficult to identify the structure of the high-pressure phase through XRD. So an absolute phase fraction of cotunnite-like phase in the sample is hard to refine by the Rietveld method. However, the intensity ratio (*I*/*I*_0_) between the scattering intensity between the (222) and (004) pyrochlore structure diffraction (*I*), which is the location of the most intense cotunnite peaks and the intensity of (222) reflection (*I*_0_), is employed as a relative phase fraction in order to determine the onset transition pressure. The *I*/*I*_0_ as a function of pressure are shown in figure 3. After approximately 40 GPa, the *I*/*I*_0_ increases rapidly, and the onset of the transition pressure is 39.8 GPa. Here, in order to compare with previous studies, the onset transition pressure is determined as 42(2) GPa based on the intensity increase of the cotunnite-like reflections.

According to group theory, pyrochlore structured Gd_1.5_Ce_0.5_Ti_2_O_7_ has 6 Raman active modes, namely,

3.1 $$\begin{array}{c} \Gamma =A_{1g}+E_{g}+4F_{2g}. \end{array}$$

Raman spectra from Gd_1.5_Ce_0.5_Ti_2_O_7_ recorded from 150 cm^−1^ to 1000 cm^−1^ at various pressures are shown in figure 2*b*. At 2.2 GPa, five vibration modes can be identified and labelled as M1 to M5. According to previous studies [18–20], M1, M4 and M5 were assigned as F_2g_ and M3 as A_g_. M2 contains two modes that are assigned as F_2g_ + E_g_ with close frequencies. The vibrations of the pressure transmitting medium (PTM) silicone oil are marked with stars (*). At 29.1 GPa, a new band appears at approximately 700 cm^−1^, and it is believed to be related to the distortion of the [TiO_6_] octahedron [21,22]. The [TiO_6_] distortion also causes the colour of the sample to change under pressure (figure 2*c*). At approximately 30 GPa, the colour of Gd_1.5_Ce_0.5_Ti_2_O_7_ changes from transparent orange-ish to dark purple-blue. Combined with the XRD pattern and Raman spectra, this colour change may be due to the distortion of the [TiO_6_] octahedron rather than the phase transition.

## 4. Discussion

**Figure 4.**
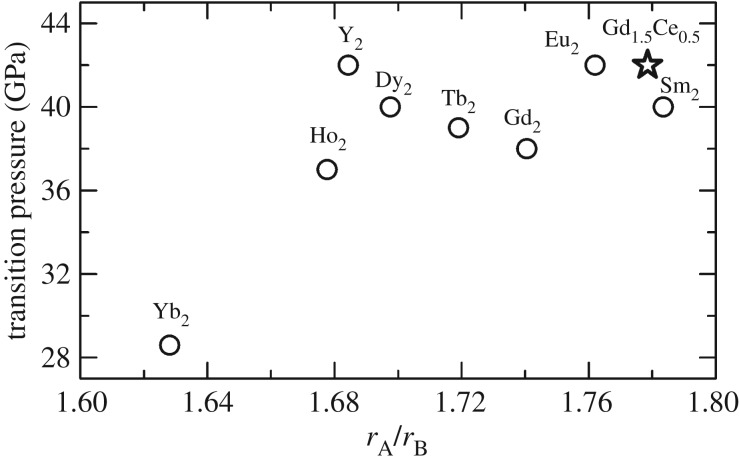
Correlation plot between the transition pressure to a cotunnite-like structure for titanite pyrochlores and the cationic radii ratio (*r*_A_/*r*_B_). The ionic radii of the A-site REE are the value of the eightfold coordinated cations with the chemical valence of +3, and Ti^4+^ ionic radius is the value of the sixfold coordinated cations. The round black marks represent the transition pressure listed from previous research (table 1) and the star represents the results of this study.

Apart from crystal structure prediction under ambient conditions, the cationic radius ratio *r*_A_/*r*_B_ is effective for predicting the high-pressure stability of pyrochlore oxides. The transition pressure of Gd_1.5_Ce_0.5_Ti_2_O_7_ is similar to that of Eu_­2_Ti_2_O_7_ [23] and Sm_2_Ti_2_O_7_ [24] but larger than that of Gd_2_Ti_2_O_7_ [11]. The transition pressures of titanite pyrochlore oxides are listed in table 1. A larger ionic radius replacement will usually lower the transition pressure. However, the current study found that as the radius of the A-site cation increased, the pyrochlore to cotunnite-like phase transition pressure rose (figure 4). This unusual tendency is related to the mechanism of the pyrochlore-cotunnite transition. The high-pressure cotunnite-like phase is a highly disordered phase. First, unlike ideal cotunnite-structured oxides (AO_2_), A and B cations are disordered in the cotunnite-like A_2_B_2_O_7_, and 1/8 of O^2−^ are randomly vacant. Second, the cotunnite-like high-pressure phase likely causes many disordered anion vacancy defects due to the vacancies present in the pyrochlore structure. From this perspective, the pressure-induced phase transition from the pyrochlore phase to the cotunnite-like phase is an order–disorder transition. When Gd^3+^ was replaced by Ce^3+^, the average cationic radius of the A site and the ratio *r*_A_/*r*_B_ increased. Theoretical calculations have also proved that pyrochlore oxide with a larger *r*_A_/*r*_B_ causes higher defect formation energy (DFE) of cation antisite and anion Frenkel defects [29]. The higher DFE hinders the order–disorder transition. The results also confirm the substitution of large cationic radius actinides in the A-site of pyrochlore oxides will increase the cationic radius ratio *r*_A_/*r*_B_, and the transition pressure. But the substitution in the B-site will decrease the stability of the pyrochlore phase by lowering the *r*_A_/*r*_B_. So during the immobilization of the high level toxic nuclear waste, the A-site substitution will obtain a more stable form.

**Table 1.** The titanite pyrochlore (A_2_Ti_2_O_7_) to cotunnite-like transition pressure versus the cationic radius ratio. The cationic radii are from Shannon, 1976 [25]. ^#^ is from the single crystal high-pressure XRD [26]. * is from the present study. –: not provided in the reference.

A-	Sm [24]	Eu [23]	Gd_1.5_Ce_0.5_*	Gd [11]	Tb [27]	Dy [23]	Y [27]	Ho [27]	Yb [28]
*r*_A_/*r*_B_	1.784	1.762	1.779	1.741	1.719	1.698	1.684	1.678	1.628
Ps (GPa)	40	42	39.8	38	39	40	42	37	28
B_0_ (GPa)	185.4(2)^#^	—	185(1)	176(4)	199(1)	—	204(3)	213(2)	219(6)
B_0_′	4.2	—	4(fixed)	6.9(1.0)	4(fixed)	—	4.2(0.2)	4(fixed)	3.2(5)

Silicate pyrochlore (B^4+^ = Si^4+^) has been synthesized under high pressure and high temperature (for example, Sc_2_Si_2_O_7_, In_2_Si_2_O_7_ and MgZrSi_2_O_7_) [30,31]. These are composed of a larger A site cation and a much smaller B site cation (Si^4+^), and their average cationic ratio is *r*_A_/*r*_B_ > 1.78. Moreover, Si–O bonds in silicate pyrochlore are more likely to form covalent bonds, which are possibly hard to break. Accompanied by the above, these silicate pyrochlore oxides should transfer to the cotunnite-like structure at a much higher pressure.

**Figure 5.**
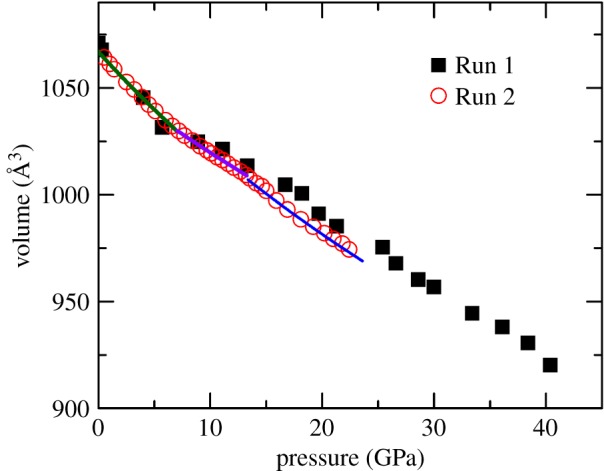
The *P*-*V* relationship of Gd_1.5_Ce_0.5_Ti_2_O_7_. The black squares are the data of run 1, with silicone oil loaded as the pressure medium. The red circles are data from run 2, whose pressure medium is noble gas argon. The *P*-*V* curves from three regions are shown in different colours: the dark green curve indicates *p* < 6.5 GPa, the purple curve shows 6.5 GPa < *p* < 13.5 GPa and the blue curve is *p* > 13.5 GPa.

**Figure 6.**
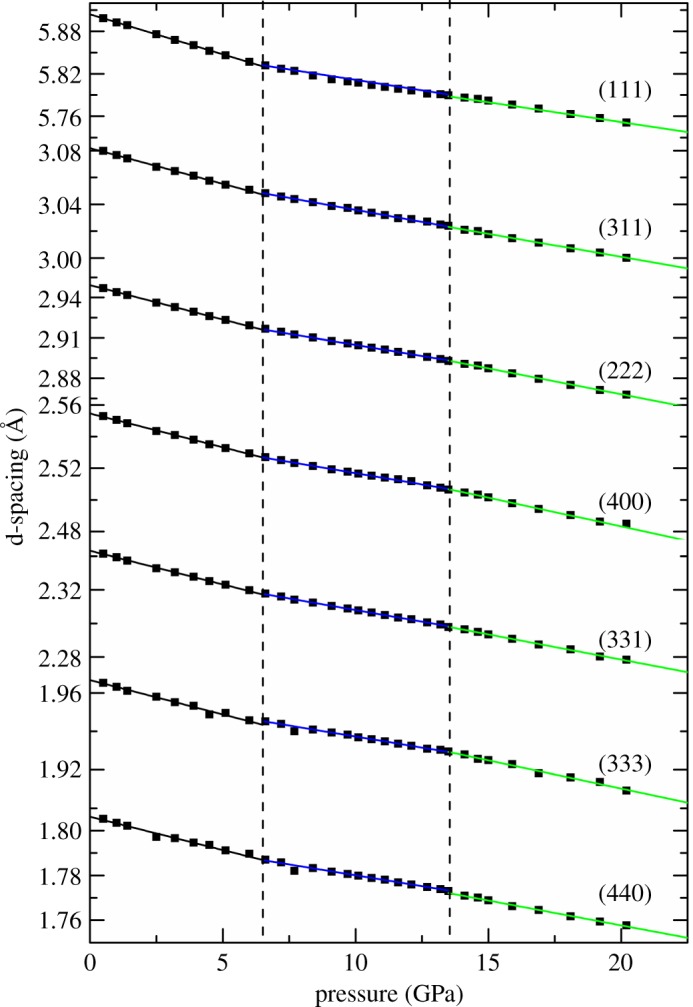
The d-spacing of Gd_1.5_Ce_0.5_Ti_2_O_7_ as a function of pressure. The black, blue and green lines are different regions (*p* < 6.5 GPa, 6.5 GPa < *p* < 13.5 GPa, and *p* > 13.5 GPa, respectively).

Structure distortion is observed at approximately 9 GPa in many other titanite pyrochlores (e.g. Gd_2_Ti_2_O_7_ [18] and Tb_2_Ti_2_O_7_ [19]). At approximately 9 GPa, the 48*f* O^2−^ moves towards the vacancies and distorts the [TiO_6_] octahedral, and this distortion is thought to be related to the pressure-induced crystallization of the spin liquid [32]. The compression behaviour of Gd_1.5­_Ce_0.5_Ti_2_O_7_ is rather complex as a function of pressure. The pressure variation of the d-spacing for some strong diffraction peaks displayed twice change in slope: 6.5 GPa and 13.5 GPa, as shown in figure 6. To obtain the unit-cell parameters of Gd_1.5_Ce_0.5_Ti_2_O_7_ at various pressures in 2 runs (run 1: *p* < 40 GPa), the Le Bail refinement based on the pyrochlore structure for the *in situ* synchrotron X-ray diffraction patterns before the transition pressure was carried out and is plotted in figure 5, and the unit-cell volumes of various pressures are listed in table 2. There are three regions in the plot with distinctly different pressure dependencies. Due to the limited number of data in run 1, 2-nd Birch-Murnaghan EoS [33] was used to fit these data only in run 2:

4.1 $$\begin{array}{c} P=\frac{3}{2}B_{0}\left[{\left(\frac{V_{0}}{V}\right)}^{7/3}-{\left(\frac{V_{0}}{V}\right)}^{5/3}\right]\times \left\{1+\frac{3}{4}({B^{\prime }}_{0}-4)\left[{\left(\frac{V_{0}}{V}\right)}^{2/3}-1\right]\right\}, \end{array}$$

where *p* is the pressure, *B*_0_ is the bulk modulus, $B_{0}^{\prime }$ is the pressure derivative of *B*_0_, *V*_0_ is the unit-cell volume at zero pressure and room temperature. $B_{0}^{\prime }$ is fitted to 4 for all of the data periods. The parameters of EoS are listed in table 3. Fitting the *P*-*V* curve before 6.5 GPa yields a bulk modulus of 185(1) GPa, which is compatible with pure Sm_2_Ti_2_O_7_, but higher than Gd_2_Ti_2_O_7_. Between 6.5 and approximately 13 GPa, the rate of change in the unit-cell volume is less than the region of *p* < 6.5 GPa, indicating an increase in the incompressibility. The *B*_0_ of this region is 261(2) GPa, which increases by 40%. At pressures higher than 13 GPa, the slope again steepens. The bulk modulus over 13 GPa decreases to 195(5) GPa. In run 1, although the pressure transmitting medium is silicone oil, the same tendency is observed. Le Bail refinement of *in situ* high-pressure XRD (electronic supplementary material, figure S2) and *in situ* high-pressure Raman spectra confirm that no phase transition occurred. The d-spacing of each hkl of pyrochlore and the a-axial length *a* at various pressures also confirmed the compressibility changes at 6.5 GPa and 13 GPa. (figure 6; electronic supplementary material, figure S2). So we think there are two isostructural changes occurring in Gd_1.5_Ce_0.5_Ti_2_O_7_ at 6.5 GPa and 13 GPa.

**Table 2.** The unit cell volumes and the *a*-axial lengths of the Gd_1.5_Ce_0.5_Ti_2_O­_7_ under different pressures from run 1 and run 2.

run 1			run 2					
*P* (GPa)	*a* (Å)	*V* (Å^3^)	*P* (GPa)	*a* (Å)	*V* (Å^3^)	*P* (GPa)	*a* (Å)	*V* (Å^3^)
0.0001	10.231(1)	1070.9(3)	0.5	10.210(1)	1064.3(2)	12.1	10.042(1)	1012.7(3)
0.3	10.221(1)	1067.8(3)	1.0	10.200(1)	1061.3(2)	12.7	10.036(1)	1010.9(4)
4.0	10.149(1)	1045.4(3)	1.4	10.192(1)	1058.8(2)	13.2	10.031(1)	1009.2(3)
5.7	10.110(1)	1031.5(4)	2.5	10.173(1)	1052.8(2)	13.5	10.026(1)	1007.7(3)
8.9	10.082(1)	1024.8(4)	3.2	10.162(1)	1049.3(2)	14.1	10.018(1)	1005.4(3)
11.1	10.070(1)	1021.3(3)	3.9	10.150(1)	1045.7(2)	14.6	10.013(1)	1004.0(3)
13.3	10.045(1)	1013.6(3)	4.5	10.139(1)	1042.2(2)	15.0	10.006(1)	1001.8(3)
16.7	10.015(1)	1004.6(3)	5.1	10.129(1)	1039.2(2)	15.9	9.991(1)	997.3(4)
18.2	10.002(1)	1000.6(4)	6.0	10.115(1)	1034.9(2)	16.9	9.977(1)	993.1(3)
19.7	9.970(1)	991.1(4)	6.6	10.106(2)	1032.2(2)	18.1	9.962(2)	988.5(3)
21.3	9.951(1)	985.3(3)	7.2	10.099(1)	1029.9(2)	19.2	9.950(1)	985.0(5)
25.4	9.917(1)	975.4(3)	7.7	10.092(1)	1027.7(2)	20.2	9.939(1)	982.0(5)
26.6	9.892(2)	967.8(5)	8.4	10.084(1)	1025.4(2)	21.0	9.931(1)	979.3(4)
28.6	9.866(1)	960.3(4)	9.1	10.075(1)	1022.7(2)	21.8	9.923(2)	977.1(4)
30.0	9.853(1)	956.8(3)	9.7	10.069(1)	1020.8(2)	22.4	9.914(1)	974.4(3)
33.4	9.811(1)	944.4(3)	10.1	10.064(1)	1019.4(2)			
36.1	9.789(1)	938.0(3)	10.6	10.059(1)	1017.6(2)			
38.4	9.763(1)	930.6(3)	11.1	10.054(1)	1016.2(2)			
40.4	9.726(1)	920.2(5)	11.6	10.048(1)	1014.5(3)

**Table 3.** The parameters of the fitted EoS of the Gd_1.5_Ce_0.5_Ti_2_O_7_ (B_0_′is fixed to 4) and the Gd_2_Ti_2_O_7_ from [18]. —: not provided in the reference.

	Gd_1.5_Ce_0.5_Ti_2_O_7_			Gd_2_Ti_2_O_7_	
*P*	0 ∼ 6.5 GPa	6.5 ∼ 13 GPa	>13 GPa	0 ∼ 8.5 GPa	>8.5 GPa
*B_0_* (GPa)	185(1)	261(2)	195(5)	176(4)	208(8)
*B_0_*′	4(fixed)			6.9(1)	1.0(3)
*V_0_* (Å^3^)	1066.9(1)	1056.8(3)	1070(2)	—	

A similar phenomenon occurs in Sm_2_Zr_2_O_7_ [34] and La_2_Zr_2_O_7_ [35], and the mechanism is thought to be related to the anion disorder. Limited to the *in situ* high-pressure XRD experiment conditions, the refined crystal structure of the Gd_1.5_Ce_0.5_Ti_2_O_7_ could not be obtained. Besides, the high-pressure Raman spectrum is also not high quality enough to obtain the vibration frequencies at various pressures because the sample studied is polycrystalline powder, so a single crystal sample is essential. On the other hand, the electron structure of Gd^3+^ is [Xe]4f^7^, and Ce^3+^ is [Xe]4f^1^, which means the Gd_1.5_Ce_0.5_Ti_2_O_7_ is undoubtedly a strong correlation system. The possibility that this complex compression behaviour is caused by the transition of the f-electron structure is hard to rule out. Finally, compared with the *P*-*V* data plotted from run 1 and run 2, the compression behaviour changes of Gd_1.5_Ce_0.5_Ti_2_O_7_ may be related to the hydrostatic condition caused by the different PTM. Although the solidification pressure is 1.4 GPa at 300 K and its hydrostatic limit is approximately 9 GPa, Ar still provides a better hydrostatic condition than silicone oil does [36]. At P below 6.5 GPa, the unit-cell volumes of the samples are in good agreement in both run 1 and run 2. At P between 6.5 GPa and 13 GPa, the slope of the P-V curve from run 1 is lower than run 2, which means in silicone oil, Gd_1.5_Ce_0.5_Ti_2_O_7_ is more incompressible than in Ar. At *P* higher than 13 GPa, the P-V curve from run 1 is systematically higher than that from run 2, possibly due to the compressibility difference in the previous pressure regions. The slope of this region from the two runs is nearly the same. So the isostructural transitions may be related to the hydrostatic conditions. Above all, elucidating the mechanism of the complex compression behaviour of Gd_1.5_Ce_0.5_Ti_2_O_7_ requires more evidence.

**Figure 7.**
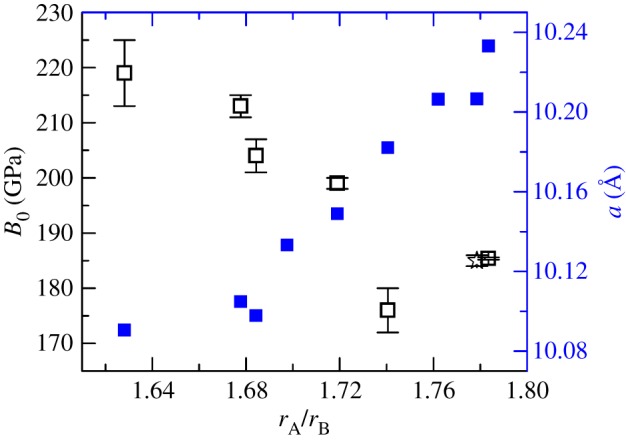
The bulk modulus of titanite pyrochlores and the unit-cell parameter *a* versus the cationic radii ratio (*r*_A_/*r*_B_). The black square marks represent the bulk modulus listed from previous research, the blue squares represent the *a* axial lengths, and the star mark represents the results of this study.

Figure 7 shows the bulk modulus (*B*_0_) when *P* < *P*_c_, (*P*_c_: the compressibility change pressure) of titanite pyrochlore oxides with different *r*_A_/*r*_B_. The bulk modulus *B*_0_ of most of the titanite pyrochlores is higher than 180 GPa. The cationic radii ratio *r*_A_/*r*_B_ is negatively correlated to the bulk modulus. This is because the smaller A-site cationic radius can shorten the bond lengths by reducing the unit-cell parameters *a*. When they are shortening, the chemical bonds will be more incompressible. The average A-site cationic radius of Gd_1.5_Ce_0.5_Ti_2_O_7_ is similar to that of Sm_2_Ti_2_O_7_ but larger than that of Gd_2_Ti_2_O_7_. So the bulk modulus of Gd_1.5_Ce_­0.5_Ti_2_O_7_ is quite similar to that of Sm­_2_Ti_2_O_7_ and higher than that of Gd_2_Ti_2_O_7_.

## 5. Conclusion

The present experimental results demonstrate that ordered pyrochlore structured Gd_1.5_Ce_0.5_Ti_2_O_7_ (*Fd*$\bar{3}$*m*, *Z* = 8) will transfer to a disordered cotunnite-like structure (*Pnma Z* = 4) at approximately 42 GPa. Compared with Gd_2_Ti_2_O_7_, 25% Gd^3+^ substituted by Ce^3+^ increases the transition pressure because the pressure-induced pyrochlore to cotunnite-like phase transition was mainly controlled by the cation order–disorder transition. Furthermore, Gd_1.5_Ce_0.5_Ti_2_O_7_'s compression behaviour is rather complex as a function of pressure. Two isostructural transitions occur at 6.5 GPa and 13 GPa, which influences the compressibility of Gd_1.5­_Ce_0.5_Ti_2_O_7,_ and the transition at 6.5 GPa may be related to the hydrostatic conditions.

## Supplementary Material

Supplementary materials

Reviewer comments

## Supplementary materials

The supplementary materials associated with this article can be found in the online version: https://doi.org/10.6084/m9.figshare.9725534.v1.

## Data accessibility

Our data are deposited at Dryad: https://doi.org/10.5061/dryad.14g7bk7 [37].

## Fundings

This work was financially supported by the National Natural Science Foundation of China (grants no. 91326102 and 51532009), and the Science and Technology Development Foundation of China Academy of Engineering Physics (grant no. 2013A0301012).
