# Supplementary material for: Pressure-induced order–disorder transition in Gd1.5Ce0.5Ti2O7 pyrochlore
Source: R Soc Open Sci. 2019 Sep 4;6(9):190842. doi: 10.1098/rsos.190842 (PMC6774973; doi:10.1098/rsos.190842)
Supplement: Supplementary materials [file rsos190842supp1.docx]

**Supplementary Materials**





Fig. S1 The selected XRD patterns of Gd_1.5_Ce_0.5_Ti_2_O­_7_ under different pressures in run2. ☆ belong to Argon’s reflections.





Fig.S2. The a-axial length *a*(Å) as a liner function of pressure. The slope change is observed as 6.5 GPa and 13 GPa, respectively.


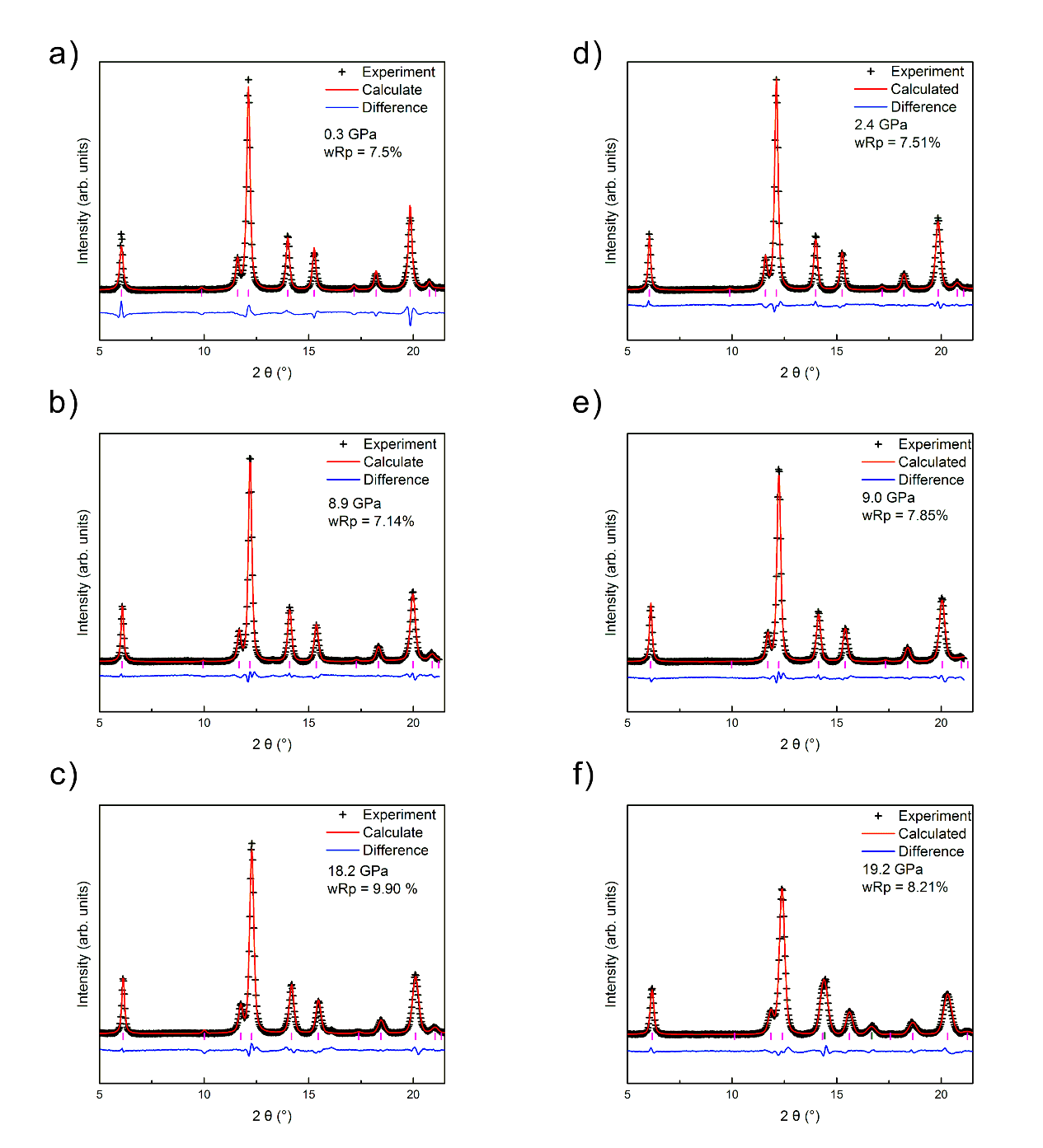


Fig. S3. Le Bail refinement of Gd_1.5­_Ce_0.5_Ti_2_O_7­_ at various pressures. a)-c) are patterns from run1, with silicone oil as pressure medium. d)-f) are patterns from run2, with Argon as the pressure medium. Magenta bar are reflections of pyrochlore structure and green bar belongs to pressure-induced solidified Argon.
